# Oxygen-Vacancy-Induced Electronic Structure Modulation in ZnTiO_3_ Perovskite: A Combined DFT and SCAPS-1D Study Toward Photovoltaic Applications

**DOI:** 10.3390/ijms27062668

**Published:** 2026-03-14

**Authors:** Angel Tenezaca, Ximena Jaramillo-Fierro

**Affiliations:** 1 Carrera de Ingeniería Química, Universidad Técnica Particular de Loja, San Cayetano Alto, Loja 1101608, Ecuador; aptenezaca1@utpl.edu.ec; 2 Departamento de Química, Facultad de Ciencias Exactas y Naturales, Universidad Técnica Particular de Loja, San Cayetano Alto, Loja 1101608, Ecuador

**Keywords:** ZnTiO_3_ perovskite, oxygen vacancies, density functional theory (DFT), SCAPS-1D simulation, perovskite oxides, bandgap narrowing, photovoltaic performance

## Abstract

Zinc titanate (ZnTiO_3_) is a chemically stable and non-toxic oxide perovskite whose photovoltaic potential remains largely unexplored due to its wide indirect bandgap. This study evaluates whether oxygen-vacancy (F-center) engineering can tailor its electronic structure and improve its suitability as a photovoltaic absorber. Density Functional Theory (DFT) calculations using VASP (PAW − GGA/PBE + U) were performed to evaluate structural stability, electronic properties, and electron affinity, while optical absorption was modeled through a combined Tauc–Gaussian approach. Device performance was assessed via SCAPS-1D simulations in an FTO/ZnO/ZnTiO_3_/Spiro-OMeTAD architecture. Oxygen vacancies induce bandgap narrowing from ~2.96 eV to ~1.47 eV and generate Ti-3d-dominated donor-like and deep intragap states. The calculated electron affinity is ~3.77 eV. Simulated single-layer devices reach Voc ≈ 1.11 V, Jsc ≈ 8.27 mA·cm^−2^, FF ≈ 83%, and a maximum efficiency of ~7.65%, primarily limited by moderate absorption strength and defect-assisted recombination. Multilayer configurations indicate that geometric optimization can significantly enhance projected efficiency, approaching 19.25% under idealized conditions. Although vacancy engineering extends visible-light absorption, the intrinsic indirect band-gap character constrains the ultimate photovoltaic performance of ZnTiO_3_.

## 1. Introduction

Zinc titanate (ZnTiO_3_) is an oxide perovskite of growing scientific and technological interest due to its chemical stability, non-toxic nature, photocatalytic activity, and tunable electronic structure enabled by defect engineering [1,2]. Compared to hybrid organic–inorganic perovskites, oxide perovskites exhibit superior structural robustness, making them promising platforms for sustainable optoelectronic applications [3,4]. In this context, perovskite-type ZnTiO_3_ has been extensively explored as an electron transport layer (ETL) owing to its favorable band alignment and high optical transparency [5]. Nevertheless, this application reflects only part of its functional potential. A more comprehensive understanding of its defect-mediated electronic structure is therefore essential to fully assess its broader optoelectronic relevance.

Zinc titanate crystallizes in several polymorphs, including ilmenite, spinel, and perovskite-type phases, whose symmetry and atomic coordination strongly influence orbital hybridization, band scattering, and defect energetics [6]. ZnTiO_3_ perovskite can adopt either hexagonal or cubic symmetry, which plays a decisive role in defining its electronic behavior. The cubic phase, characterized by greater symmetry, facilitates electron redistribution and can promote higher carrier mobility. Conversely, the hexagonal phase generally exhibits greater thermodynamic stability and lower susceptibility to structural collapse following the formation of an oxygen vacancy [6,7]. Overall, ZnTiO_3_ perovskite behaves as a wide-bandgap nonmagnetic semiconductor with predominantly indirect transitions. The valence band (VB) is governed mainly by O-2p states, while the conduction band (CB) is dominated by Ti-3d orbitals, with additional contributions from Zn-3d states [8,9].

Intrinsic point defects, particularly oxygen vacancies, critically influence the electronic response of oxide perovskites, including ZnTiO_3_. Neutral oxygen vacancies (F-centers) introduce localized states within the bandgap and modify charge redistribution, symmetry, and electrostatic alignment [10]. Depending on their energetic position and orbital hybridization, these states may act as shallow donor-like levels that increase carrier density or as deep traps that promote non-radiative recombination [7]. In titanate systems, vacancy formation is often accompanied by Ti-centered electronic localization. This process leads to band-gap renormalization and defect-induced optical absorption [11]. Consequently, oxygen-vacancy control provides a strategy for tuning the photophysical properties of ZnTiO_3_. However, this strategy increases the complexity of device optimization and requires computational methods capable of resolving defect–electronic interactions with atomic precision [12].

From an optical perspective, absorption in ZnTiO_3_ has traditionally been described using Tauc-based models combined with Gaussian functions to represent sub-gap states associated with intrinsic defects or structural disorder [2]. These models reproduce macroscopic spectral features, including indirect absorption-slopes and Urbach tails. However, they do not fully capture electronic transitions mediated by O-2p and Ti-3d interactions or the redistribution of defect-induced states in wide-bandgap systems [13]. This limitation highlights the need to complement classical optical models with ab initio electronic-structure calculations that explicitly account for vacancy-induced orbital modulation.

Recent advances in perovskite solar cell (PSC) research have shown that computational tools such as VASP and SCAPS provide powerful frameworks for linking fundamental material properties to device-level performance. Density Functional Theory (DFT) enables the evaluation of structural stability, free energies, density of states, and defect energetics with a level of resolution that is difficult to achieve experimentally [14]. SCAPS has become a standard platform for one-dimensional photovoltaic modeling. It allows the assessment of charge transport, trap densities, band alignment, and interfacial effects in multilayer architectures [15,16]. Even in DFT may underestimate bandgaps [17] and drift–diffusion simulations involve geometric idealizations [14], the combined use of these methods provides a coherent structure–property–function framework for emerging photovoltaic materials.

Although PSC technologies have reached efficiencies comparable to crystalline silicon, large-scale deployment remains limited by long-term instability and lead toxicity [18]. These challenges motivate the exploration of stable inorganic alternatives, including oxide-based perovskites. However, a comprehensive molecular-level analysis that integrates structural phase stability, oxygen-vacancy energetics, orbital-resolved electronic states, vacuum-level alignment, and device-relevant parameters has not yet been systematically developed for ZnTiO_3_. In particular, the extent to which vacancy-induced bandgap narrowing and Ti-3d state redistribution modify electron affinity, optical absorption, and macroscopic photovoltaic response remains insufficiently clarified.

In this study, oxygen vacancy-induced modulation of the electronic structure in perovskite-like ZnTiO_3_ was investigated using first-principles DFT calculations combined with one-dimensional photovoltaic simulations. Phase stability, vacancy stabilization energies, orbital-resolved density of states, band-gap renormalization, and electrostatic alignment were analyzed. The results elucidate how neutral F centers redistribute Ti-3d-derived states and reshape the electronic landscape. The resulting electronic parameters were incorporated into SCAPS simulations to evaluate their impact on photovoltaic performance in an FTO/ZnO/ZnTiO_3_/Spiro-OMeTAD architecture. This integrated approach provides molecular-level insight into defect physics in ZnTiO_3_ and clarifies its realistic potential within emerging oxide-based photovoltaic materials.

## 2. Results and Discussion

### 2.1. Structural Optimization of ZnTiO_3_

**Figure 1:**
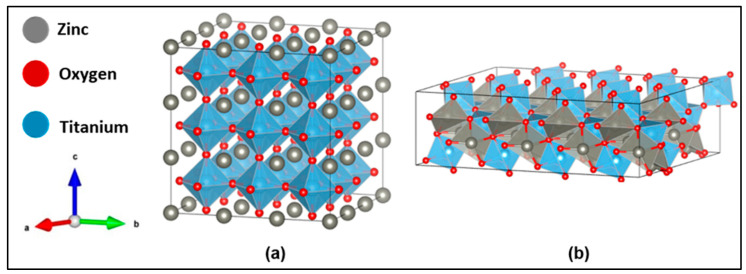
Optimized structures of supercells of ZnTiO_3_: (**a**) cubic and (**b**) hexagonal.

Structural optimization of ZnTiO_3_ was performed in the cubic and hexagonal phases to establish a consistent reference frame before electronic and optical analyses. The cubic supercell of ZnTiO_3_ (Figure 1a) converged to lattice parameters of a = b = c = 11.58 Å and angles α = β = γ = 90°, while the hexagonal phase was modeled using its crystallographically equivalent trigonal representation (Figure 1b), which offers a compact, symmetry-preserving description of the lattice without compromising structural fidelity. This choice is supported by previous studies demonstrating that the R-3 hexagonal phase can be rigorously mapped onto a rhombohedral/trigonal lattice [8,19] whose supercell relaxed to a = 14.86 Å, b = 20.59 Å, c = 6.50 Å, with angles α = β = 90° and γ = 99.9763° [20].

The fractional atomic coordinates of the cubic and hexagonal ZnTiO_3_ supercells are presented in Tables S1 and S2, respectively. On the other hand, Table S3 presents the total free energies obtained for bulk and slab configurations of ZnTiO_3_ in the hexagonal and cubic phases after full structural relaxation. These values serve as a reference for comparing the relative stability of the models considered. For the cubic phase, only bulk and pristine slab configurations are reported, exhibiting higher total free energies than their hexagonal counterparts. On this basis, the hexagonal structure was selected as the reference phase for the defect analysis and for the electronic, optical, and device-level simulations discussed in the main text.

**Figure 2:**
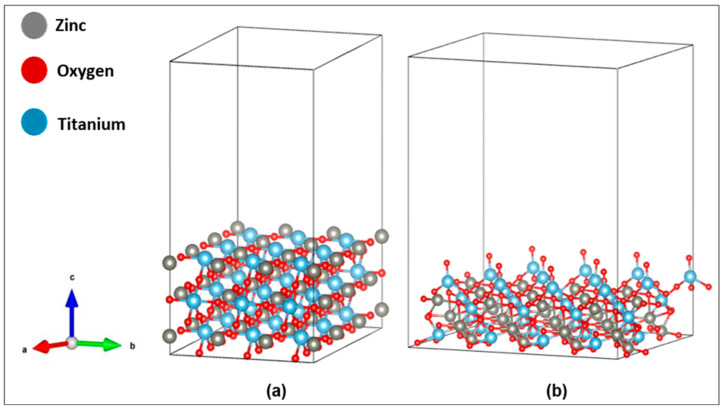
Optimized structures of plane (101) of ZnTiO_3_ perovskite: (**a**) cubic and (**b**) hexagonal.

The surface models presented in Figure 2 were constructed from the optimized supercells shown in Figure 1 by cleaving the bulk along the (101) crystallographic plane and periodically extending the slab in the in-plane directions to generate equivalent surfaces for the cubic and hexagonal polymorphs of ZnTiO_3_. Each slab consisted of three atomic layers and included a 15 Å vacuum region along the surface normal to suppress false interactions between periodic images and to ensure an unambiguous reference for electrostatic potential.

The decision to investigate only the (101) plane is based on thermodynamic and application-relevant considerations, as several DFT studies have shown that the (101) facet exhibits the lowest surface energy among the accessible terminations of structures derived from ZnTiO_3_ and related titanates, and is therefore considered the most stable and representative surface under ambient or near-ambient conditions [20]. Therefore, this study does not re-evaluate the stability of ZnTiO_3_ surfaces; instead, it focuses on the electronic and defect-related properties of the most well-supported stable (101) slab, quantifying its response to oxygen-vacancy formation and assessing its relevance for device-level band alignment.

### 2.2. Determination of Electronic Properties of ZnTiO_3_

Table 1 compiles reported cases of bandgap reduction in the range of titanates and oxides following the introduction of oxygen vacancies (VO). These values provide a baseline overview that helps anticipate the degree to which ZnTiO_3_ might be affected under comparable defect conditions. For each compound, the table contrasts the pristine bandgap with the value obtained after VO creation, highlighting the characteristic narrowing observed across different systems. In this context, only neutral oxygen vacancies (referred to as F centers) are considered, as they represent the most physically stable defect state and are widely reported under intrinsic or near-stoichiometric conditions [2,6,7]. Charged vacancies are highly dependent on the Fermi-level position, extrinsic doping, and specific electrochemical environments, introducing additional variables that complicate direct comparison between different materials [21].

From the data summarized in Table 1, the average band-gap reduction induced by the neutral oxygen vacancies in titanates and related oxides is approximately 1.09 eV, with reported values ranging from 0.70 to 1.50 eV. This range reflects the vacancy-driven band-gap reduction obtained for ZnTiO_3_ in this study, with the gap narrowing from 2.96 eV to ~1.48 eV (Table 2), yielding ΔE ≈ 1.48 eV. This confirms that the defect-induced electronic restructuring observed here is within the expected physical regime for oxide perovskites, thus validating the use of obtained values.

**Figure 3:**
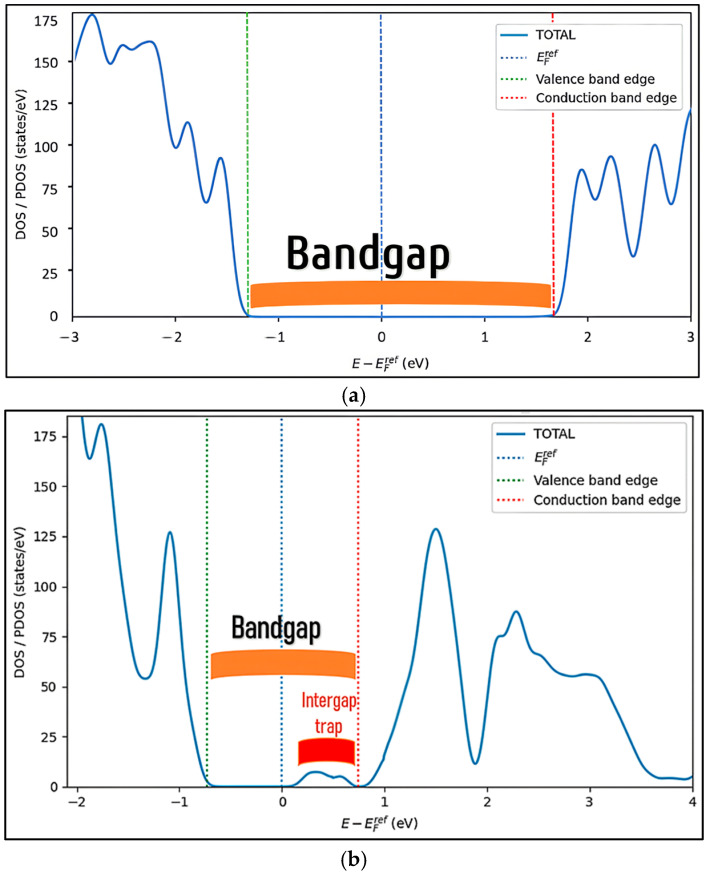
Density of states (DOSs) of ZnTiO_3_: (**a**) without vacancies and (**b**) with vacancies.

In the defect-free case, (Figure 3a) the DOS exhibits a well-defined forbidden region with no electronic states within the bandgap, which is consistent with the wide-gap semiconductor nature of ZnTiO_3_. Following the introduction of oxygen vacancies (Figure 3b), the DOS profile is characterized by an apparent narrowing of the bandgap and the appearance of localized intragap states. These intragap features originate from F centers associated with the oxygen vacancies [27] and perform two electronic roles. States located near the conduction band minimum act as surface donor levels, effectively supplying free electrons and facilitating n-type conductivity. Conversely, deeper states, located farther from the band edges, behave as deep traps, capturing charge carriers and acting as recombination centers [28]. This differentiation between donor-like states and deep traps is fundamental, as it explains why oxygen vacancies simultaneously enhance optical absorption through narrowing of the bandgap and, at the same time, introduce recombination pathways that can limit the carrier’s lifetime if its density becomes excessive.

**Figure 4:**
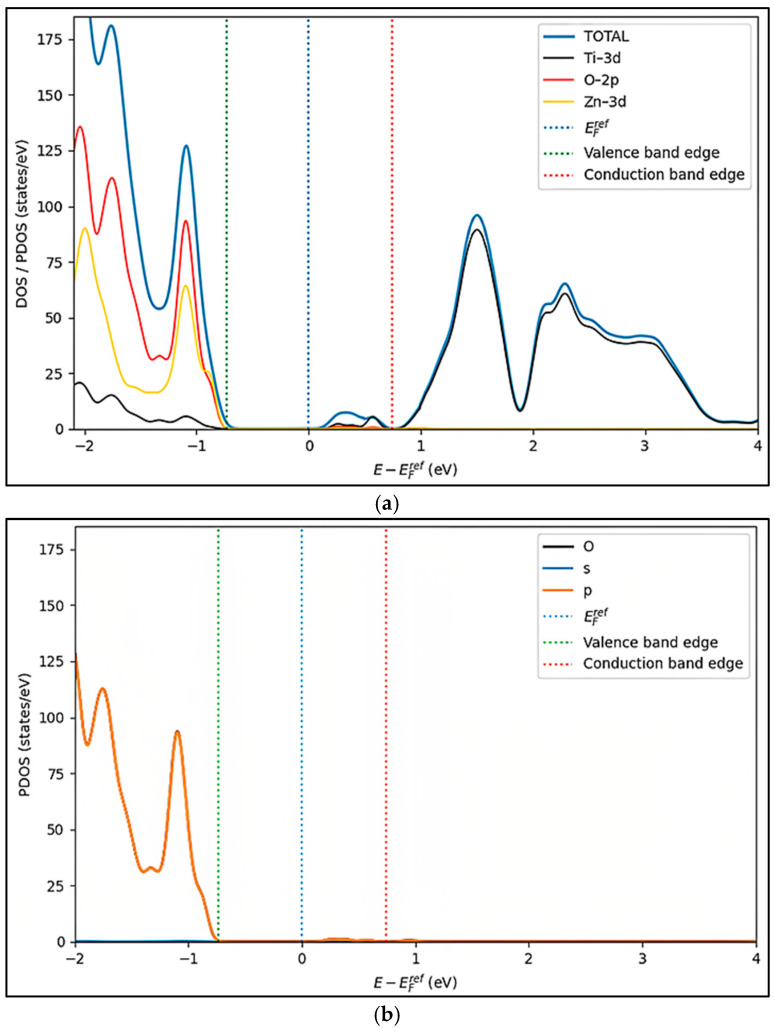

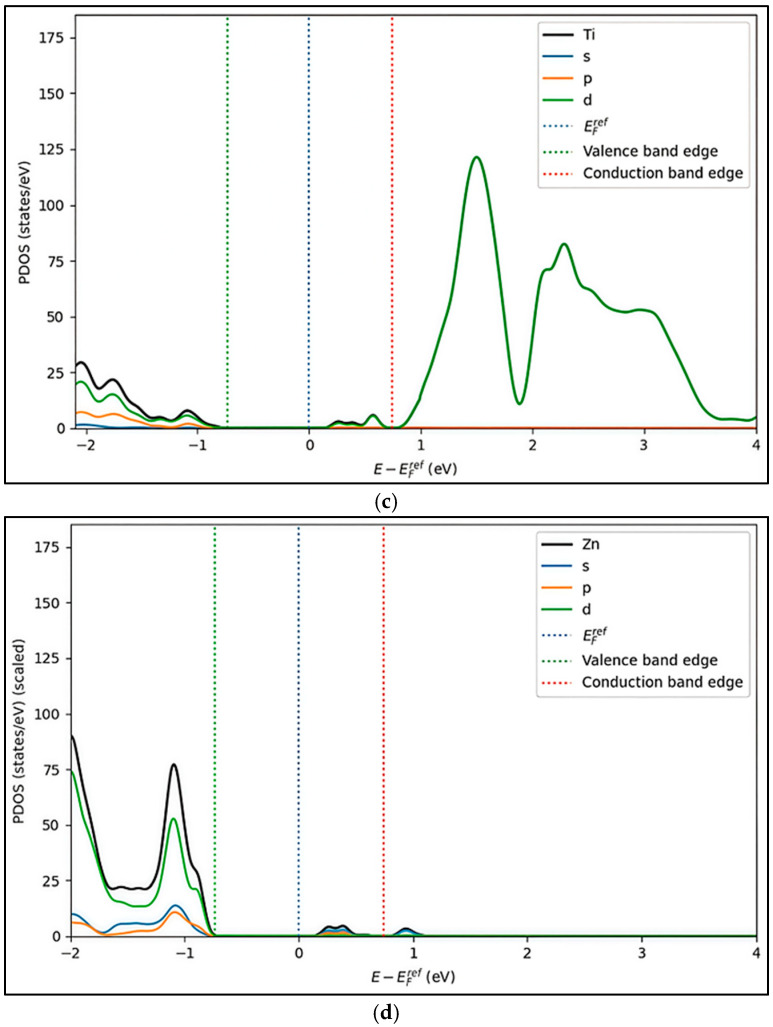
Density of states (DOSs) of ZnTiO_3_: (**a**) total and partial of (**b**) O, (**c**) Ti and (**d**) Zn.

In the present study, the density-of-states analysis reveals that the electronic states induced by the vacancy are predominantly located near the conduction band. This energetic positioning indicates that oxygen vacancies in ZnTiO_3_ primarily exhibit donor-like behavior under neutral charge conditions, effectively contributing electrons to the conduction band. As shown in the total (Figure 4a) and partial DOS, the valence-band region is predominantly governed by O-2p states with additional hybridization from Zn-3d orbitals, while the conduction-band edge is largely dominated by Ti-3d states, which agrees with previous electronic-structure analyses of ZnTiO_3_ [20]. It is evident that removing the oxygen suppresses the O-2p contribution near the valence-band edge (Figure 4b) and increases the Ti-3d weight near the conduction-band minimum. This occurs because the excess electrons released by the oxygen vacancy are accommodated in Ti-centered states [7]. The partial DOS bandgap (Figure 4c) is predominantly governed by Ti-3d contributions. Near the conduction-band edge, Ti-3d-derived states are energetically shallow and delocalized, behaving as donor-like levels, thereby increasing the availability of electrons for n-type conduction [29]. At lower energies within the gap, Ti-3d states become progressively more localized, with reduced hybridization with O-2p orbitals, resulting in deep features within the gap that act as efficient carrier trapping and recombination centers [29]. The absence of significant contributions derived from Zn near the conduction-band edge (Figure 4d) further confirms the secondary electronic role of Zn in carrier transport, reinforcing that the vacancy-driven electronic restructuring is primarily mediated by the Ti–O sublattice [6].

**Figure 5:**
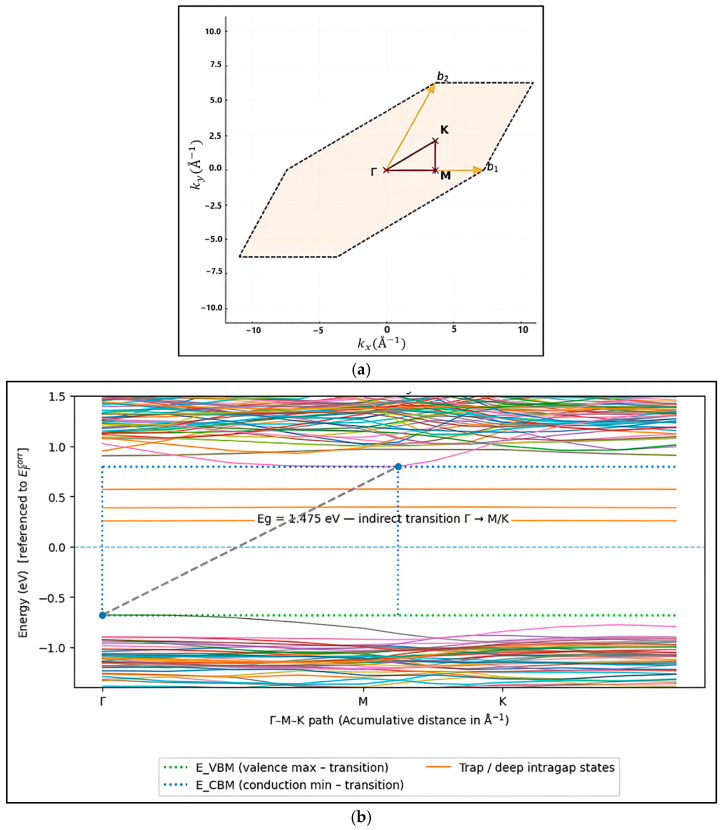
(**a**) Two-dimensional representation of the first Brillouin zone for the hexagonal primitive cell used in the band structure calculation, with high-symmetry k-points (Γ, M and K) and the corresponding reciprocal vectors indicated (b_1_ and b_2_). (**b**) Band structure of ZnTiO_3_ along the high-symmetry Γ–M–K path in the first Brillouin zone.

The electronic band structure calculated along the high-symmetry Γ–M–K path (Figure 5a) provides further insight into the defect-mediated electronic behavior of ZnTiO_3_. The adoption of a two-dimensional (2D) reference path within the basal plane of the Brillouin zone is justified by the hexagonal/ilmenite-like symmetry of the stabilized phase, for which the most relevant band ends and dispersion features are captured by k-space directions [30]. As evidenced in Figure 5b, the material exhibits an indirect band-gap transition, with the valence-band maximum located at the Γ region and the conduction-band minimum shifted toward the M/K region, resulting in an effective bandgap of approximately 1.47–1.48 eV; this value is consistent with that previously obtained by the density-of-states (DOS) analysis. Furthermore, the appearance of nearly flat bands within the forbidden gap, characterized by low k-dispersion, indicates the presence of highly localized defect states (deep traps) [29]. Conversely, the bands forming the edge of the conduction-band display a higher dispersion, indicating lower effective electron masses and higher carrier mobility, which supports the donor-like role of oxygen vacancies inferred from the DOS results [8]. Overall, the coexistence of dispersive conduction states and localized intragap levels, induced by oxygen vacancies, simultaneously causes an improvement in electron mobility and Shockley–Read–Hall Recombination (SRH), which affect photovoltaic efficiency [29].

### 2.3. Determination of the Absorption Model of ZnTiO_3_ Perovskite

Density-of-states and band structure analyses demonstrate that F centers generate both donor-like Ti-3d states and deeper, localized intragap states [7]. This lowers the energy threshold for allowed optical transitions, extending the absorption edge into the visible spectral range [31]. However, despite the theoretical evidence supporting defect-induced absorption in perovskites and mixed oxides [32], direct experimental determination of the absorption coefficient for ZnTiO_3_ under controlled oxygen-vacancy concentrations is limited. Most experimental studies on ZnTiO_3_ have focused on photocatalytic performance, dielectric properties, or optical transparency in the UV region, without addressing the contribution of vacancy-induced subgap states to visible-light absorption [3]. In the absence of experimental evidence indicating that oxygen vacancies significantly enhance the absolute magnitude of the absorption coefficient, the optical response of ZnTiO_3_ (plus vacancies) was conservatively set at the absorption level reported for pristine ZnTiO_3_ (α ≈ 10^4^ cm^−1^) [31], while the vacancy-induced bandgap narrowing regulates the shift in the absorption onset. Consequently, the absorption model adopted in this study is based on electronic structure DFT calculations to establish a physically consistent optical response.

**Figure 6:**
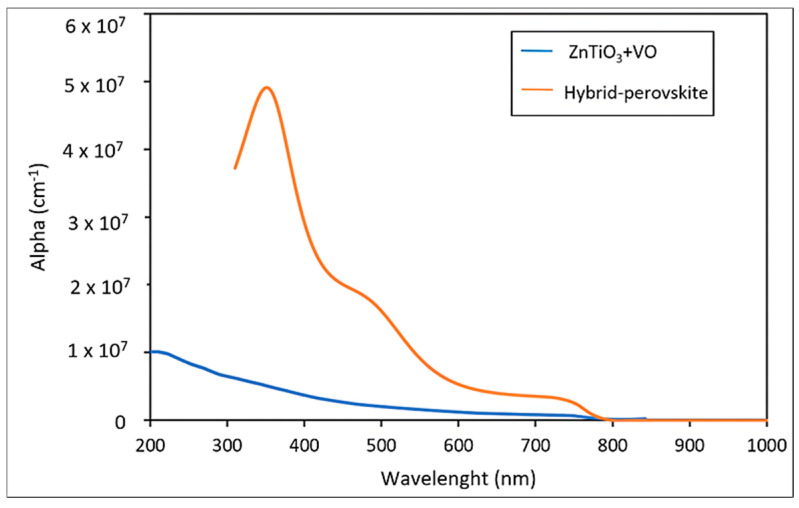
Optical absorption curves of ZnTiO_3_ perovskite and hybrid-perovskite (CH_3_NH_3_PbI_3_).

Comparison with the hybrid halide perovskite CH_3_NH_3_PbI_3_, currently considered the reference absorber for high-efficiency perovskite solar cells, provides further physical insight into the implications of the obtained absorption model. As shown in Figure 6, the hybrid perovskite exhibits absorption coefficients on the order of 10^5^ cm^−1^ across most of the visible spectrum, while ZnTiO_3_ shows values approximately an order of magnitude lower. This pronounced difference arises from the distinct optical mechanisms governing each material: in hybrid perovskites, strong absorption originates from direct bandgap transitions and highly dispersive band edges [2]. In contrast, ZnTiO_3_ retains an indirect bandgap character even after vacancy-induced narrowing, which intrinsically limits transition probabilities and reduces the absorption strength [6]. This comparison shows that ZnTiO_3_ (enhanced with defect engineering) does not achieve absorption levels comparable to those of hybrid perovskites, but it offers superior stability, a non-toxic composition, and an adjustable electronic structure. Therefore, rather than competing with hybrid perovskites solely in terms of optical power, ZnTiO_3_ is better suited for use as a complementary absorber or in tandem and multilayer architectures, where these characteristics can be leveraged to improve the overall performance of the device.

### 2.4. Analysis of Photovoltaic Performance of ZnTiO_3_ with F-Centers Using SCAPS-1D

The photovoltaic performance of ZnTiO_3_ was evaluated in a standard perovskite-type solar cell architecture using the SCAPS-1D numerical simulator. The objective of this analysis was to establish a baseline evaluation of the photovoltaic behavior of ZnTiO_3_ as an absorber layer, rather than seeking exhaustive optimization of the device configuration. Therefore, a conventional FTO/ZnO/ZnTiO_3_/Spiro-OMeTAD structure was adopted, employing electron and hole transport layers, which are widely used in perovskite solar cells and whose electronic properties are well documented in the literature [33]. This choice allows the role of ZnTiO_3_ within a reproducible framework, while minimizing the influence of factors such as advanced interface engineering or material-specific optimization strategies, which are outside the scope of this initial study.

The physical and electronic parameters assigned to each layer, including thickness, bandgap, electron affinity, dielectric permittivity, effective density of states, carrier mobilities, and doping concentrations, are summarized in Table 3. These values were chosen based on previously determined electronic properties for ZnTiO_3_ and on parameter sets commonly reported for Fluorine-doped tin oxide (FTO), ZnO, and Spiro-OMeTAD in SCAPS-based simulations of perovskite solar cells [16]. For the ZnTiO_3_ absorber, the modeled defects do not explicitly correspond to oxygen vacancies but rather represent generic bulk defect states distributed throughout the material that can act as surface or deep recombination centers, consistent with the electronic structure analysis discussed in the previous sections [16,28]. Only SRH recombination was considered in the present simulations. Radiative recombination was ruled out due to the indirect bandgap of ZnTiO_3_, whose radiative transitions are weak and do not dominate carrier losses under standard operating conditions [34]. Auger recombination, although potentially relevant at very high carrier densities [29], was not included to avoid unnecessary model complexity and additional fitting parameters. While its inclusion could increase the realism of the simulation, it would divert attention from the main objective of this study, which is to provide a practical and physically consistent estimate of the expected photovoltaic performance of ZnTiO_3_.

**Figure 7:**
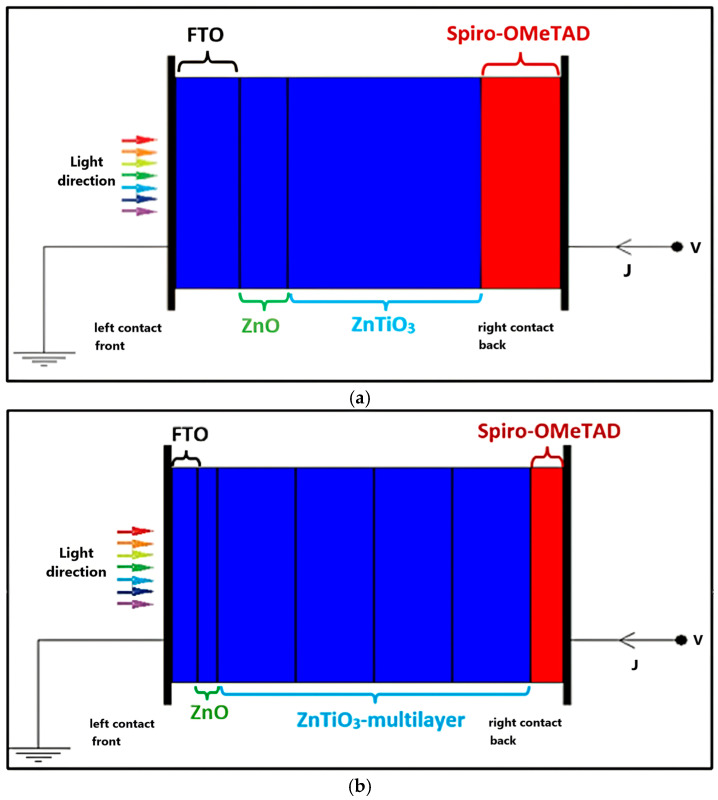
Scheme of ZnTiO_3_-type perovskite solar cell: (**a**) single-layer (feasible model) and (**b**) multilayer (hypothetical model). In the figure, blue layers represent n-type regions, and the red layers represent p-type regions. Besides, V denotes the applied voltage at the back contact, while J represents the current density flowing through the device.

Figure 7 illustrates the device configurations considered for the SCAPS simulations. The single-layer structure shown in Figure 7a represents the physically feasible reference model, in which ZnTiO_3_ is considered a homogeneous absorber layer. In contrast, the multilayer configuration depicted in Figure 7b corresponds to a hypothetical construct introduced to explore the effect of spatially distributed defect populations and varying donor–trap ratios along the absorber thickness.

The configuration shown in Figure 7 is not intended to reproduce a viable manufacturing route but rather serve as a numerical framework for examining how different defect fractions influence carrier screening, recombination behavior, and overall device performance. Based on this approach, the volumetric densities of deep trap states and donor-like states were systematically varied according to the relative fraction of oxygen vacancies, as summarized in Table 4. For each vacancy concentration, the total effective defect density N_t_ was kept constant, while the relative contribution of the deep traps and donor states was adjusted. This allows for a clearer interpretation of how the donor–trap equilibrium governs the electrostatic sensing length and recombination dynamics within the absorber.

The Debye length L_D_ provides a direct measure of the length of electrostatic shielding within the absorber and is therefore a key criterion for device stability. To maintain an effective electric field in the depletion region, the absorber thickness *z* must be several times greater than the Debye length, thus satisfying the empirical condition: L_D_ < $\frac{z}{5}$, which means that the L_D_ must remain below approximately 15–25% of the total thickness [37,38].

Experimental depth profiling studies in perovskite oxides and related oxide semiconductors indicate that vacancy redistribution and near-surface electrostatic effects typically extend to several tens of nanometers from the surface [39], defining a near-surface defect-modulated region. Similarly, space charge layer widths in SrTiO_3_-based defective systems have been reported to range from tens to hundreds of nanometers, depending on the doping level and thermodynamic conditions [40]. Considering these experimentally observed spatial scales, an absorber thickness of approximately 250–300 nm was selected. This range ensures that the vacancy-affected domain near the surface is fully contained, while allowing the remaining portion of the film to approach bulk-like behavior. Beyond 300 nm, the exponential decay of defect concentration progressively reduces donor states and deep traps to marginal levels, typically well below 0.01% of the surface value, such that ZnTiO_3_ progressively approaches bulk-like behavior and vacancy-induced visible absorption is effectively suppressed [7].

It should be noted that determining the exact characteristic thickness of the evaluated material lies beyond the scope of this study. Therefore, the values adopted are considered a physical reference scale, based on experimentally reported magnitudes in comparable oxide systems. Under this configuration, satisfying the empirical electrostatic condition of the total thickness implies a characteristic shielding length on the order of 30–60 nm. This leads to the adoption of a representative decay length of 50 nm for the spatial distribution of vacancy-induced perturbations. It is worth highlighting that, although the present DFT calculations consider neutral oxygen vacancies (F-centers), the characteristic length adopted here reflects the spatial scale at which defect-induced electronic perturbations and electrostatic shielding effects coexist in the device environment.

Following the criterion, Debye lengths below ≤50 nm were identified as the desirable regime, as they allow adequate penetration of the internal electric field maintaining efficient carrier transport and defect-assisted optical absorption [41]. As shown in Table 4, this condition is only met in donor-dominated configurations with vacancy fractions of 0.10% and 0.05%, where the Debye length (L_D_) remains in the range of ~31–49 nm depending on the relative balance between donor states and deep traps. Conversely, a vacancy fractions of 0.01% produce Debye lengths well above this threshold, indicating an over-screened regime in which electrostatic field penetration is strongly suppressed and device performance becomes electrostatically limited. For this reason, the configuration with θvo = 0.01% was not considered in the photovoltaic simulations summarized in Table 5, since such conditions would lead to electrostatically limited device operation rather than a representative working regime of the absorber layer.

The photovoltaic behavior of the device as a function of the vacancy percentage and the relative fractions of the donor and trap states are shown in Table 5. Across all the tested vacancy concentrations and donor–trap ratios, the short-circuit current density (J_sc_) exhibits negligible variation, indicating that the photocurrent is primarily governed by the intrinsic optical absorption of ZnTiO_3_ and is largely insensitive to changes in the defect distribution within the explored range [16]. In contrast, the open-circuit voltage (V_oc_) shows a clear dependence on the donor–trap balance. Configurations characterized by higher donor-state fractions yield improved voltage output, which can be attributed to a reduction in non-radiative losses governed by Shockley–Read–Hall (SRH) recombination, resulting in longer effective carrier lifetimes and a reduced recombination rate under open-circuit conditions [42]. The fill factor (FF) exhibits moderate variations as the defect distribution changes; this produces changes in carrier transport efficiency, along with variations in the effective strength of SRH recombination within the absorber layer [28]. Deep-level defects enhance carrier capture and recombination, increasing resistive losses and altering the shape of the current–voltage curve [38], while donor-dominated configurations enhance electrostatic shielding and charge harvesting by reducing the Debye length and strengthening the internal electric field [4].

As reflected in the photovoltaic parameters reported in Table 5, the highest power conversion efficiencies (η) for the ZnTiO_3_-based device were 7.65%. While this value represents a favorable balance between donor states and deep traps within the explored parameter space, it remains limited by the absorption-driven constraint on photocurrent generation. In this respect, the multilayer configuration should be considered a theoretical upper-bound scenario, illustrating the extent to which geometric effects can partially mitigate optical losses without altering the underlying electronic structure of the absorber.

**Figure 8:**
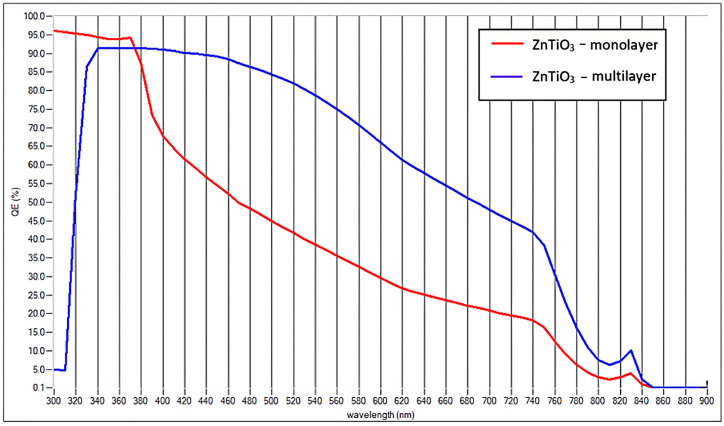
Variation of EQE for ZnTiO_3_: multilayer and single layer.

The spectral response of the ZnTiO_3_-based devices is illustrated in Figure 8, which compares the external quantum efficiency (EQE) of the monolayer and multilayer configurations. In the monolayer architecture, the EQE exhibits a rapid decline beyond the near-UV region, reflecting the limited absorption capability of ZnTiO_3_ in the visible spectrum. This behavior is directly related to the material’s indirect bandgap and its moderate absorption coefficient, which restricts efficient photogeneration to a relatively narrow wavelength range [6,29]. Consequently, carrier generation at longer wavelengths is significantly reduced, imposing a fundamental limitation on the achievable short-circuit current density. In contrast, the theoretical multilayer configuration shows a remarkably broader EQE response extending further into the visible region, indicating a higher probability of photon absorption across the device. The multilayer configuration represents an idealized scenario designed to explore the potential impact of geometric light-management strategies on ZnTiO_3_-based devices. In this architecture, the absorber is redistributed into several sublayers, effectively increasing the optical path length without modifying the intrinsic electronic structure of the material. As a result, the probability of photon absorption within the device increases, partially compensating for the relatively low absorption coefficient characteristic of ZnTiO_3_. It is important to emphasize that the higher projected efficiency obtained for the multilayer configuration does not arise from changes in the vacancy-induced electronic structure or from variations in the donor–trap equilibrium analyzed for the single-layer system. As shown in Table 5, the simulated efficiency exhibits only minor variations with the vacancy fraction within the explored range (θvo = 0.05–0.10%), indicating that the overall device performance is primarily controlled by the donor–trap balance rather than by the absolute vacancy concentration. The multilayer configuration was therefore evaluated under the defect-engineering conditions that yielded the best performance in the single-layer device, corresponding to a donor-dominated configuration (θ_D_ = 0.9 and θ_tr_ = 0.1) and a representative vacancy fraction of θvo = 0.07%. Under these conditions, the efficiency enhancement observed in the multilayer architecture originates mainly from the improved light harvesting enabled by thickness scaling and geometric redistribution of the absorber. By dividing the absorber into thinner sublayers, the effective optical path length increases while maintaining favorable carrier collection conditions [43]. Consequently, the multilayer configuration should be interpreted as a theoretical upper-bound scenario illustrating the maximum performance that ZnTiO_3_ could achieve if its intrinsic optical limitations were partially mitigated through advanced device design, allowing geometric light-management effects to be distinguished from vacancy-induced electronic modifications.

Table 6 presents a comparative analysis of the devices presented in this study with established photovoltaic technologies.

As Table 6 shows, ZnTiO_3_-based devices within the broader photovoltaic landscape, enabling a direct comparison with established technologies. While the single-layer configuration exhibits clearly limited efficiency (7.65%), the multi-layer architecture achieves projected values of 19.25% with an idealized thickness scaling (≈2 µm). At this level, ZnTiO_3_ surpasses the typical efficiencies of amorphous silicon cells and approaches those of multicrystalline silicon technologies; however, it still falls short of the performance achieved by next-generation hybrid halide perovskites, perovskite-silicon tandem cells, and advanced heterojunction architectures. The superior efficiencies reported for these systems arise primarily from their direct or quasi-direct bandgap nature and substantially higher absorption coefficients, which enable efficient photogeneration across a broad spectral range using absorber layers that are approximately an order of magnitude thinner [48,49,50]. Furthermore, their more favorable charge transport properties and lower sensitivity to defect-mediated recombination facilitate the simultaneous achievement of high short-circuit current densities and open-circuit voltages [5]. Consequently, while multilayer structuring and vacancy engineering improve the performance of ZnTiO_3_, its intrinsic optical limitations cannot be fully compensated for by thickness scaling or geometric light management strategies alone.

## 3. Materials and Methods

### 3.1. Computational Methodology

All electronic and structural calculations were performed using Density Functional Theory (DFT) as implemented in the Vienna Ab initio Simulation Package (VASP), version 6.2.1, developed by VASP Software GmbH in Vienna, Austria [51,52]. Molecular modeling and visualization were performed using BioVia Materials Studio, version 5.5, provided by BioVia in San Diego, CA, USA. The interaction between valence electrons and ionic cores was described using the projector augmented wave (PAW) method [53], while the exchange-correlation effects were treated within the generalized gradient approximation (GGA) employing the Perdew–Burke–Ernzerhof (PBE) functional [54,55]. The Kohn–Sham equations were solved self-consistently using a plane-wave basis set with a kinetic energy cutoff of 500 eV [56]. Structural optimizations were performed until the electronic self-consistency criterion reached and energy change below $10^{-5}$ eV and the residual Hellmann–Feynman forces were smaller than 0.005 eV·Å^−1^ [57]. Brillouin-zone integrations were conducted using the Monkhorst–Pack scheme [58], with k-point meshes adapted to each optimization stage; for slab geometries, the adequacy of the k-point sampling was then verified using k-gamma and k-spacing criteria.

Primitive cells were optimized using symmetric k-point meshes of 3 × 3 × 3 (cubic) and 3 × 2 × 1 (hexagonal). Slab models were then generated by applying a (101) crystallographic cut to both phases, introducing a vacuum region of 15 Å along the surface-normal direction. Selective dynamics were applied to the two outermost oxygen layers, and a 3 × 2 × 1 k-point mesh was employed for all slab calculations. The formation energy of a single neutral oxygen vacancy (F center) was evaluated at different surface and subsurface positions within the slab in order to identify the energetically most favorable configuration. Spin polarization and an initial magnetic moment distributed over the two Ti atoms neighboring the vacancy were considered [21]. Once the optimal vacancy configuration was identified, electronic-structure calculations were refined by incorporating the Hubbard on-site correction (+U). Different U values were examined, yielding an optimal combination of U = 5.00 eV for Ti and U = 7.25 eV for Zn [7].

### 3.2. Determination of Electronic and Optical Properties of ZnTiO_3_

The electronic band structure was computed along high-symmetry paths in the reduced two-dimensional Brillouin zone (Γ–M–K–Γ) [30,59,60]. Total and partial densities of states (DOS and PDOS) were calculated to identify the orbital contributions of the valence and conduction bands, as well as to assess the impact of structural phase and defect formation on the electronic structure. The macroscopic electrostatic potential was extracted along the surface-normal direction to determine the vacuum reference level [61]. A linear correction was applied in the vacuum region to remove the residual slope, and the vacuum level was defined from the most stable plateau (right-hand side), ensuring a field-free reference [57,62,63]. Based on this corrected alignment, the electron affinity (*χ*) was calculated as the difference between the vacuum level and the conduction-band minimum referenced at the center of the slab [6]:

(1) $$\chi =E_{vac}-E_{CB}^{center}$$

where E_vac_ represents the corrected vacuum level, defined as the planar-averaged electrostatic potential in the vacuum region after removal of the artificial slope, and $E_{CB}^{center}$ denotes the conduction band minimum (CBM) in the central, bulk-like region of the slab, sufficiently far from both vacuum interfaces to avoid surface or electrostatic artifacts.

Optical properties such as dielectric permittivity (ε) were derived from the complex dielectric function obtained within the independent-particle approximation (IPA) [64]. Within this framework, microscopic local-field effects (LFE) were not explicitly included since their incorporation requires evaluation of the full microscopic dielectric matrix, which substantially increases the computational cost, particularly for large supercells and slab geometries containing defects, as those considered in the present study [65]. It is worth noting that neglecting LFE within the IPA may influence the quantitative accuracy of the calculated dielectric response, especially in systems exhibiting strong microscopic charge inhomogeneity or reduced symmetry [66]. This limitation can be more pronounced in slab geometries, where the presence of vacuum interfaces and defect-induced charge redistribution may enhance local electrostatic variations near the surface [67]. Nevertheless, for wide-bandgap oxide semiconductors such as ZnTiO_3_, the IPA framework can still provide useful qualitative insight into the optical response, including the approximate position of the absorption edge and the effect of defect-induced states on optical transitions. Consequently, the present optical model should be interpreted as providing a first-order approximation of the absorption behavior of defect-engineered ZnTiO_3_ rather than a complete many-body description of its optical response [65]. On other hand, since slab geometries include a finite vacuum fraction that dilutes the dielectric response, a correction was applied based on the ratio between the slab thickness and the total supercell length along the surface-normal direction [68]. The effective high-frequency dielectric constant was extracted from the corrected out-of-plane component, $\varepsilon_{\infty ,zz}$, which is appropriate for non-polar slab geometries [65].

### 3.3. Parameters for Photovoltaic Performance Evaluation

After establishing the electronic properties of ZnTiO_3_, its photovoltaic performance was evaluated through device-level simulations using the one-dimensional Solar Cell Capacitance Simulator (SCAPS-1D), version 3.3.11 developed at Department of Electronics and Information Systems (ELIS) of the University of Gent, Belgium [16]. The simulations were performed under steady-state conditions by solving the coupled Poisson and carrier continuity equations within the drift–diffusion [16,69]. The electrostatic potential is obtained from Poisson’s equation [29]:

(2) $$\frac{d^{2}\psi (x)}{dx^{2}}=-\frac{q}{\varepsilon }\times [p\left(x\right)-n\left(x\right)+N_{D}^{+}\left(x\right)-N_{A}^{-}(x)]$$

where $\frac{d^{2}\psi (x)}{dx^{2}}$ represents the spatial curvature of the electrostatic potential, q and ε correspond to the elementary charge and the permittivity of the material, respectively, p and n refer to concentration of holes and electrons, respectively, and $N_{D}^{+}\left(x\right)$ and $N_{A}^{-}(x)$ denote the concentration of ionized donor and acceptor impurities, respectively. Carrier transport was described by continuity equations [29]:

(3) $$\nabla \cdot J_{n}=q(G\left(x\right)-R\left(x\right))$$

(4) $$\nabla \cdot J_{p}=-q(G\left(x\right)-R\left(x\right))$$

where $G\left(x\right)$ is the carrier generation, $R\left(x\right)$ is the carrier recombination rate (including trap-assisted channels intrinsic to the SRH-limited regime considered in this study), and $J_{n}$ and $J_{p}$ are the electron and hole drift–diffusion current densities, which are defined as [16]:

(5) $$J_{n}=qn\mu_{n}E+qD_{n}\nabla n$$

(6) $$J_{p}=qn\mu_{p}E-qD_{p}\nabla p$$

where $\mu_{n}$ and $\mu_{p}$ correspond to the mobility of electrons and hole, E is the electric field inside the material, ∇n and ∇p represent the spatial concentration gradient of electron and hole concentration, respectively, and $D_{n}$ and $D_{p}$ are the diffusion coefficient of electrons and holes.

Because the optical response directly determines the photogeneration rate and, consequently, the attainable short-circuit current density $J_{SC}$, the absorption coefficient was modeled using a hybrid analytical parametrization. The formulation combines an indirect Tauc-type onset for phonon-assisted transitions that is representative of pristine ZnTiO_3_ and anchored to its characteristic UV absorption magnitude [2,70], with a Gaussian sub-gap contribution accounting for vacancy-induced intragap transitions, as defined by [71,72,73]:

(7) $$\alpha_{total}=\frac{1}{t_{slab}}\int_{0}^{t_{slab}}\left[\alpha_{Tauc}(E,E_{g}\left(z\right))+\alpha_{def}(E)\right]dz$$

where $t_{slab}$ represents the thickness of the ZnTiO_3_ absorber layer (300 nm), $\alpha_{Tauc}$ corresponds to the interband (band-to-band) absorption component, and $\alpha_{def}$ denotes the defect-induced absorption associated with centers F (oxygen vacancies).

The Tauc component follows the conventional quadratic energy dependence for indirect semiconductors and is implemented here in a scaled form to accommodate the selected broadening parameters [74,75]:

(8) $$\alpha_{Tauc}\;\;\left(E,E_{g}(z)\right)=\frac{\alpha_{E04}}{{\left(∆E\right)}^{2}}\times (E-E_{g}(z){)}^{2},E\geq E_{g}$$

where E denotes incident photon energy, and $E_{g}(z)$ represents the effective indirect optical bandgap of the material, which may vary with depth due to bandgap narrowing. The parameter ∆E is an energy scaling parameter introduced to control the effective amplitude of the indirect onset, such that $E_{04}=E_{g}+∆E$, and $\alpha_{E04}=10^{4}$  cm^−1^ defines the calibration condition, ensuring that the absorption coefficient reaches the prescribed reference magnitude at the energy $E_{04}$. The prefactor therefore acts as an effective normalization term that enforces this amplitude constraint while preserving the quadratic energy dependence characteristic of indirect transitions. Notably, the value $\alpha_{E04}$ was adopted as the reference magnitude for pristine ZnTiO_3_, in line with previously reported optical properties [6,70].

Oxygen vacancies introduce localized electronic states and modify the host electronic structure, producing defect-induced band-gap renormalization. Because the formation energy of oxygen vacancies increases with depth, their concentration is expected to decrease away from the surface, generating a defect-modulated region near the surface followed by a progressively bulk-like region as the thickness increases [7,76]. As a consequence, the magnitude of the defect-induced bandgap renormalization is not spatially uniform within the absorber layer, and the optical bandgap progressively approaches the pristine value at larger depths where the vacancy concentration becomes negligible. To represent this physical behavior in the optical absorption model, the bandgap was described as a depth-dependent function $E_{g}(z)$, assuming that the local band-gap renormalization scales with the local vacancy concentration. This formulation provides an effective description of the gradual transition between the defect-rich surface region and the bulk-like interior of the absorber. Accordingly, the bandgap variation along the depth was expressed as [37,77,78]:

(9) $$E_{g}\left(z\right)=E_{g,0}-{∆E}_{g}^{BGN}\left(z\right)=E_{g,0}-{BGN}_{max}(\frac{C_{VO}(z)}{C_{0}})$$

where $E_{g,0}$ denotes the pristine bandgap of ZnTiO_3_ (i.e., in the absence of vacancies), and ${BGN}_{max}$ represents the maximum band-gap reduction, defined as ${BGN}_{max}=E_{g,0}-E_{g,VO}$, with $E_{g,VO}$ corresponding to the bandgap at the vacancy-rich surface. The term $C_{VO}(z)$ describes the local concentration of oxygen vacancies, assumed to follow an exponential decay profile, which is in agreement with previously reported near-surface defect gradients [6,79]:

(10) $$C_{VO}\left(z\right)=C_{0}\times e^{-\frac{z}{L}}$$

Here, $C_{0}$ is the reference vacancy concentration, and L denotes the effective decay length that governs the spatial distribution of defects. In this study, L was set to 50 nm, defining the characteristic length scale over which the vacancy concentration decreases. Substituting Equation (10) into Equation (9) results in the cancellation of $C_{0}$, so that the band-gap renormalization depends solely on the characteristic ratio α/L and becomes independent of the absolute magnitude of the initial vacancy concentration.

On the other hand, to capture defect-enabled sub-gap absorption, the vacancy-related contribution was modeled as a Gaussian oscillator [75,80], given by the equation [81]:

(11) $$\alpha_{def}\left(E\right)=\alpha_{max}\times \exp\left[-\frac{{\left(E-E_{0}\right)}^{2}}{2\sigma^{2}}\right]$$

where $\alpha_{max}$ represents the maximum amplitude of the defect-induced absorption, $E_{0}$ is the central energy of the gaussian oscillator and σ is the standard deviation (energy broadening parameter) of the Gaussian, which is mathematically related to the full width at half maximum (FWHM) through identity $\sigma =\frac{FWHM}{2.355}$. The center energy $E_{0}=0.85eV$ was selected to position the defect-enabled sub-gap absorption band in the near-infrared (λ≈1.46 μm), i.e., well below the intrinsic interband absorption edge of ZnTiO_3_. This ensures that the added term captures weak intragap or defect-related transitions without distorting the band-edge fit. It is worth emphasizing that in optical dispersion modeling of defected oxide semiconductors, additional Gaussian oscillators are incorporated in the sub-eV regime (up to ~0.8 eV) to account for defect-induced sub-gap absorption features [82,83,84].

The peak magnitude of the defect-related Gaussian contribution was set to $\alpha_{\max}=5\;\times 10^{4}$ cm^−1^. This value maintains the physically meaningful sub-gap absorption while not exceeding the intrinsic interband absorption predicted by the Tauc model, whose maximum remains below 1 × 10^5^ cm^−1^ in the present model. By restricting the defect-related amplitude to about half of the intrinsic maximum, the model maintains band-to-band absorption as the dominant optical process, while the Gaussian contribution simply extends absorption into the sub-gap region without redefining the absorption threshold. The Gaussian broadening parameter was defined as σ = 0.60 eV which corresponds to FWHM = 1.41 eV. This broadening falls within the range of Gaussian widths reported in optical dispersion studies of perovskite-type defected oxides and TiO_x_, where FWHM values on the order of 1–2 eV are commonly used to reproduce distributed sub-gap absorption features [71,85]. In this study, vacancy concentrations on the order of 10^−3^ and 10^−4^ per lattice site were considered, corresponding to moderately high but non-degenerate defect densities. Such defect densities generate deep traps that lead to inhomogeneous energetic distributions resulting from local structural distortion, variations in coordination geometry, and defect–defect interactions [72,86,87]. Finally, a minimum optical background of 100 cm^−1^ was imposed to avoid non-physical vanishing absorption at the lowest energies [2,31].

Bulk and interface defect states were incorporated in the SCAPS simulations using a simplified Shockley–Read–Hall description, assuming neutral single-level traps with symmetric electron and hole capture cross-sections (Table 7). For all layers, bulk defect states were referenced with respect to the nearest band edge (below the conduction band for n-type layers and above the valence band for p-type layers), with characteristic energy offsets selected to represent shallow-to-intermediate recombination centers. In the ZnTiO_3_ absorber, the modeled bulk defect does not explicitly correspond to oxygen vacancies but rather represents an effective distribution of intrinsic defects within the bulk that may act as shallow or deep SRH recombination centers [16,28].

Interface defects were treated independently, with lower defect densities than in the bulk and energy levels positioned relative to the local band alignment at each interface, reflecting their role as non-ideal recombination sites rather than dominant transport-limiting centers (Table 8). The adopted defect densities and capture cross-sections were selected to ensure numerical stability and physical consistency, while allowing the recombination dynamics to be governed primarily by the absorber layer and its defect-mediated electronic structure.

## 4. Conclusions

This study demonstrated that perovskite-type ZnTiO_3_, particularly in its hexagonal phase, exhibits superior structural stability relative to the cubic phase and preserves its crystalline integrity under controlled oxygen-vacancy formation. Density Functional Theory (DFT) calculations confirmed that neutral oxygen vacancies act predominantly as donor-like defects, inducing significant bandgap narrowing from approximately 2.96 eV to ~1.47 eV while maintaining the intrinsic indirect band-gap character of the material. Density-of-states analysis revealed the coexistence of shallow Ti-3d–derived donor states and deeper localized trap states, clarifying the dual role of vacancies in enhancing carrier density while simultaneously introducing recombination pathways.

From an optoelectronic standpoint, the combined Tauc–Gaussian absorption model consistently described the vacancy-induced extension of the absorption edge into the visible region. However, even under optimized vacancy fractions and absorber thickness, ZnTiO_3_ retains a moderate absorption coefficient compared to direct-bandgap semiconductors, limiting the achievable photogeneration rate. SCAPS simulations within the FTO/ZnO/ZnTiO_3_/Spiro-OMeTAD architecture showed that device performance is maximized within a narrow donor-dominated regime, yielding Voc ≈ 1.11 V, FF ≈ 83%, and a power conversion efficiency of approximately 7.65% for the single-layer configuration.

Importantly, the multilayer configuration explored in this work indicates that geometric and thickness-scaling strategies can substantially enhance the projected efficiency, approaching 19.25% under idealized conditions. While this value does not reach the performance levels of state-of-the-art hybrid perovskites or tandem architectures, it places ZnTiO_3_ within a technologically relevant efficiency window comparable to certain commercial silicon-based technologies. These findings suggest that, although intrinsic optical constraints associated with the indirect bandgap impose an upper performance limit, multilayer structuring and defect engineering enable ZnTiO_3_ to approach moderate efficiency regimes without altering its fundamental electronic structure.

Overall, ZnTiO_3_ does not emerge as a competitive high-efficiency absorber for single-junction photovoltaic applications in its current form. Nevertheless, its structural robustness, non-toxic composition, and tunable defect-mediated electronic properties indicate potential for alternative photovoltaic strategies, including multilayer or hybrid architectures where stability and defect tolerance are prioritized. Future experimental validation and advanced light-management designs will be essential to determine whether the theoretical upper-bound performance identified here can be realistically approached. In addition, more rigorous optical modeling incorporating many-body effects, such as excitonic interactions described within the Bethe–Salpeter framework, would allow a more accurate description of the optical spectra beyond the independent-particle approximation adopted in this work.

## Figures and Tables

**Table 1:** Modification of bandgap energy (E_g_) due to F-centers.

Compound	E_g_ Original (eV)	E_g_ with VO (eV)	Difference ΔE	Reference
Lead Titanate	3.200	2.360	0.840	[11]
Barium Titanate	1.787	1.000	0.787	[22]
Strontium Titanate	3.200	1.895	1.305	[23]
Calcium Titanate	3.550	2.050	1.500	[24]
Anatase (TiO_2_)	3.100	1.940 − 1.500	1.160	[25]
Zinc Oxide (ZnO)	3.200	2.500	0.700	[26]

**Table 2:** Effect of the F-center on electronic levels and bandgap.

Structure	Fermi Level (eV)	Conduction Band Level (eV)	Valence Band Level (eV)	Bandgap (eV)
ZnTiO_3_ without vacancy	3.098	1.661	−1.296	2.957
* ZnTiO_3_–slab 101 with one vacancy	−2.884	0.780	−0.695	1.475

* The electronic levels of the slab with a vacancy include the dipolar plateau correction.

**Table 3:** Input parameters in SCAPS for each layer of the PSC.

Parameter	FTO [35,36]	ZnO (ETL) [33]	ZnTiO_3_	Spiro-OMeTAD (HTL) [36]
Thickness (nm)	500	50	300	100
Bandgap E_g_ (eV)	3.5	2	1.475	3.00
Electron affinity χ (eV)	4.0	4.00	3.770	2.45
$\mathrm{Dielectric}\;\mathrm{permittivity}\;(\varepsilon_{r})$	9.0	9.0	10.50	3.00
Effective density of states in CB, $N_{c}\;{(cm}^{-3})$	$2.2\times 10^{18}$	$3.7\times 10^{18}$	$2.2\times 10^{18}$	$2.2\times 10^{18}$
Effective density of states in BV, $N_{v}\;({cm}^{-3})$	$1.8\times 10^{19}$	$1.8\times 10^{19}$	$1.8\times 10^{19}$	$1.8\times 10^{19}$
Thermal velocity of electrons $\left(cm\;s^{-1}\right)$	$1.0\times 10^{7}$	$1.0\times 10^{7}$	$1.0\times 10^{7}$	$1.0\times 10^{7}$
Thermal velocity of holes $\left(cm\;s^{-1}\right)$	$1.0\times 10^{7}$	$1.0\times 10^{7}$	$1.0\times 10^{7}$	$1.0\times 10^{7}$
Electron mobility, μ_n_ (cm^2^ V^−1^ s^−1^)	20	100	2.5	$2\times 10^{-4}$
Hole mobility, μ_p_ (cm^2^ V^−1^ s^−1^)	10	25	1.0	$2\times 10^{-4}$
$\mathrm{Donor}\;\mathrm{density},\;N_{D}\;({cm}^{-3})$	$2.0\times 10^{19}$	$5.0\times 10^{17}$	Variable	0
$\mathrm{Acceptor}\;\mathrm{density},\;N_{A}\;({cm}^{-3})$	0	0	0	$2.0\times 10^{18}$

**Table 4:** Volumetric densities of deep traps and donor states as function of relative fractions for different oxygen vacancy concentrations.

Vacancies Fraction θ_VO_ (%)	Deep Trap Fraction θ_tr_	Donor Fraction θ_D_	Deep Trap Density N_tr_ (cm^−3^)	Donor Density N_D_ (cm^−3^)	Debye Length L_D_ (nm)
0.1 (1 VO for every 1000 crystal sites) $N_{t,eff}$ (${cm}^{-3}$) = 1.77 × 10^16^	0.5	0.5	8.85 × 10^15^	8.85 × 10^15^	41.18
	0.4	0.6	7.08 × 10^15^	1.06 × 10^16^	37.59
	0.3	0.7	5.31 × 10^15^	1.24 × 10^16^	34.80
	0.2	0.8	3.54 × 10^15^	1.42 × 10^16^	32.56
	0.1	0.9	1.77 × 10^15^	1.59 × 10^16^	30.69
0.05 (1 VO for every 5000 crystal sites) $N_{t,eff}$ (${cm}^{-3}$) = 8.85 × 10^15^	0.5	0.5	4.43 × 10^15^	4.43 × 10^15^	58.24
	0.4	0.6	3.54 × 10^15^	5.31 × 10^15^	53.16
	0.3	0.7	2.66 × 10^15^	6.20 × 10^15^	49.22
	0.2	0.8	1.77 × 10^15^	7.08 × 10^15^	46.04
	0.1	0.9	8.85 × 10^14^	7.97 × 10^15^	43.41
0.01 (1 VO for every 10,000 crystal sites) $N_{t,eff}$ (${cm}^{-3}$) = 1.77 × 10^15^	0.5	0.5	8.85 × 10^14^	8.85 × 10^14^	130.22
	0.4	0.6	7.08 × 10^14^	1.06 × 10^15^	118.88
	0.3	0.7	5.31 × 10^14^	1.24 × 10^15^	110.06
	0.2	0.8	3.54 × 10^14^	1.42 × 10^15^	102.95
	0.1	0.9	1.77 × 10^14^	1.59 × 10^15^	97.06

**Table 5:** Analysis of photovoltaic cell performance as a function of vacancy percentage and donor-trap fractions. θ_VO_: oxygen-vacancy fraction; θ_tr_: deep-trap fraction; θ_D_: donor fraction; N_tr_: deep-trap density; N_D_: donor density; Voc: open-circuit voltage; Jsc: short-circuit current density; FF: fill factor; η: power conversion efficiency.

$\theta_{VO}$ (%)	$\theta_{tr}$	$\theta_{D}$	N_tr_ (cm^−3^)	N_D_ (cm^−3^)	$V_{oc}$ (V)	J_sc_ ($\frac{mA}{{cm}^{2}}$)	FF (%)	η (%)
0.05	0.9	0.1	7.97 × 10^15^	88.85 × 10^14^	1.0999	8.268539	83.63	7.61
	0.7	0.3	6.20 × 10^15^	2.66 × 10^15^	1.1029	8.268556	83.48	7.61
	0.6	0.4	5.31 × 10^15^	3.54 × 10^15^	1.1045	8.268564	83.40	7.62
	0.5	0.5	4.43 × 10^15^	4.43 × 10^15^	1.1063	8.268572	83.33	7.62
	0.4	0.6	3.54 × 10^15^	5.3 × 10^15^	1.1081	8.268580	83.26	7.63
	0.3	0.7	2.66 × 10^15^	6.20 × 10^15^	1.1101	8.268588	83.18	7.64
	0.1	0.9	8.85 × 10^14^	7.97 × 10^15^	1.1146	8.268605	82.98	7.65
0.07	0.9	0.1	1.06 × 10^16^	1.18 × 10^15^	1.114	8.268514	83.82	7.60
	0.7	0.	8.26 × 10^15^	3.54 × 10^15^	1.0997	8.268536	83.65	7.61
	0.6	0.4	7.08 × 10^15^	4.72 × 10^15^	1.1017	8.268547	83.55	7.61
	0.5	0.5	5.90 × 10^15^	5.90 × 10^15^	1.1037	8.268558	83.45	7.62
	0.4	0.6	4.72 × 10^15^	7.08 × 10^15^	1.1059	8.268569	83.36	7.62
	0.3	0.7	3.54 × 10^15^	8.26 × 10^15^	1.1083	8.26858	83.27	7.63
	0.1	0.9	1.18 × 10^15^	1.06 × 10^16^	1.1139	8.268602	83.03	7.65
0.10	0.9	0.1	1.59 × 10^16^	1.77 × 10^15^	1.0918	8.268458	84.07	7.59
	0.7	0.3	1.24 × 10^16^	5.31 × 10^15^	1.0943	8.268496	83.92	7.59
	0.6	0.4	1.06 × 10^16^	7.08 × 10^15^	1.0967	8.268513	83.81	7.60
	0.5	0.5	8.85 × 10^16^	8.85 × 10^15^	1.0994	8.268529	83.68	7.61
	0.4	0.6	7.08 × 10^16^	1.06 × 10^16^	1.1022	8.268545	83.55	7.61
	0.3	0.7	5.31 × 10^15^	1.24 × 10^16^	1.1053	8.268562	83.41	7.62
	0.1	0.9	1.77 × 10^15^	1.59 × 10^16^	1.1127	8.268595	83.11	7.65

**Table 6:** Comparison of the external quantum efficiency (EQE) of ZnTiO_3_ with respect to other photovoltaic materials.

Solar Cell Type	Efficiency (%)	Reference
ZnTiO_3_ perovskite single layer with VO	7.65	This work
ZnTiO_3_ perovskite multilayer with VO	19.25	This work
Doped or nanomaterial-modified PSC	16.00	[18]
Advanced commercial PSC	25.60	[44]
PSC with deep molecular passivation	25.95	[44,45]
Inorganic PSC (RbGeI_3_)	23.00	[14]
Hybrid halide-organic perovskite	25.05	[18]
Perovskite–Si tandem cells	29.50	[44,45]
Bifacial PERC/HJT cells	23.50	[43]
HIT/HJT cells	26.20	[43]
Amorphous silicon cells	11.00	[46]
Multicrystalline silicon cells	20.00	[47]
Monocrystalline silicon cells	23.50	[43]

**Table 7:** Modeling parameters for PSC layer defects.

Parameter	Spiro-OMeTAD [35]	FTO [36]	ZnO [33]	* ZnTiO_3_ (Active Layer)
Defect type	Neutral	Neutral	Neutral	Neutral
Electron capture cross-section (cm^2^)	$1.0\times 10^{-15}$	$1.0\times 10^{-15}$	$1.0\times 10^{-15}$	$1.0\times 10^{-15}$
Hole capture cross-section (cm^2^)	$1.0\times 10^{-15}$	$1.0\times 10^{-15}$	$1.0\times 10^{-15}$	$1.0\times 10^{-15}$
Energy distribution	Single	Single	Single	Single
Reference for defect level (E_t_)	Above EV	Below EC	Below EC	Below EC
Energy with respect to reference (eV)	0.400	0.100	0.400	0.400
Defect density (cm^−3^)	$1.0\times 10^{14}$	$1.0\times 10^{14}$	$1.0\times 10^{14}$	$5\times 10^{13}$

* The defect modeled for ZnTiO_3_ does not correspond to oxygen vacancies, but rather to other defects that may be distributed within the bulk (forming shallow or deep SRH traps).

**Table 8:** Modeling parameters for PSC interfaces.

Parameter	FTO/ETL [88]	ETL/ZnTiO_3_ [88]	ZnTiO_3_/HTL [69]
Defect type	Neutral	Neutral	Neutral
Electron capture cross-section (cm^2^)	$1.0\times 10^{-19}$	$1.0\times 10^{-19}$	$1.0\times 10^{-19}$
Hole capture cross-section (cm^2^)	$1.0\times 10^{-19}$	$1.0\times 10^{-19}$	$1.0\times 10^{-19}$
Energy distribution	Single	Single	Single
Reference for defect level (E_t_)	Above middle of interface gap	Below the lowest EC	Above the highest EV
Energy with respect to reference (eV)	0.100	0.100	0.100
Defect density (cm^−3^)	$1.0\times 10^{12}$	$1.0\times 10^{12}$	$1.0\times 10^{12}$

## Data Availability

The original contributions presented in this study are included in the article and Supplementary Material. Further inquiries can be directed to the corresponding author.

## Supplementary Materials

The following supporting information can be downloaded at: https://www.mdpi.com/article/10.3390/ijms27062668/s1.

## Abbreviations

The following abbreviations are used in this manuscript:

DFT	Density Functional Theory
SCAPS	Solar Cell Capacitance Simulator
VASP	Vienna Ab initio Simulation Package
VO	Oxygen Vacancy
SRH	Shockley-Read-Hall Recombination
DOS	Density of states
PDOS	Projected Density of States
PSC	Perovskite Solar Cell
ETL	Electron Transport Layer
HTL	Hole Transport Layer
FTO	Fluorine-doped tin oxide
Spiro-OMeTAD	2,2′,7,7′-tetrakis(N,N-di-p-methoxyphenylamine)-9,9′-spirobifluorene
EQE	External Quantum Efficiency
GGA	Generalized Gradient Approximation
PBE	Perdew–Burke–Ernzerhof functional
IPA	Independent-particle approximation
LFE	Local-field effects
Eg	Bandgap energy
CBM	Conduction band minimum
VBM	Valence band maximum
ND	Donor density
Ntr	Trap density
Voc	Open-circuit voltage
Jsc	Short-circuit current density
FF	Fill factor
η	Power conversion efficiency
