# Supplementary material for: Oxygen-Vacancy-Induced Electronic Structure Modulation in ZnTiO3 Perovskite: A Combined DFT and SCAPS-1D Study Toward Photovoltaic Applications
Source: Int J Mol Sci. 2026 Mar 14;27(6):2668. doi: 10.3390/ijms27062668 (PMC13027390; doi:10.3390/ijms27062668)
Supplement: Supplementary file 1 [file ijms-27-02668-s001.zip › ijms-4200308-supplementary.pdf]

International Journal of
Molecular Sciences

MDPI

Supplementary Materials

Oxygen-Vacancy-Induced Electronic Structure Modulation in $ZnTiO_3$ Perovskite: A Combined DFT and SCAPS-1D Study Toward Photovoltaic Applications

Angel Tenezaca [1], and Ximena Jaramillo-Fierro [2,*]

1 Carrera de Ingeniería Química. Universidad Técnica Particular de Loja. San Cayetano Alto. Loja. 1101608. Ecuador; aptenezaca1@utpl.edu.ec
2 Departamento de Química. Facultad de Ciencias Exactas y Naturales. Universidad Técnica Particular de Loja. San Cayetano Alto. Loja. 1101608. Ecuador; xvjaramillo@utpl.edu.ec
* Correspondence: xvjaramillo@utpl.edu.ec ; Tel.: +593-7-3701444

Abstract

Zinc titanate ($ZnTiO_3$) is a chemically stable and non-toxic oxide perovskite whose photovoltaic potential remains largely unexplored due to its wide indirect bandgap. This study evaluates whether oxygen-vacancy (F-center) engineering can tailor its electronic structure and improve its suitability as a photovoltaic absorber. Density Functional Theory (DFT) calculations using VASP (PAW–GGA/PBE +U) were performed to evaluate structural stability, electronic properties, and electron affinity, while optical absorption was modeled through a combined Tauc–Gaussian approach. Device performance was assessed via SCAPS-1D simulations in an FTO/ZnO/$ZnTiO_3$/Spiro-OMeTAD architecture. Oxygen vacancies induce band-gap narrowing from ~2.96 eV to ~1.47 eV and generate Ti-3d–dominated donor-like and deep intragap states. The calculated electron affinity is ~3.77 eV. Simulated single-layer devices reach $V_{oc} \approx 1.11$ V, $J_{sc} \approx 8.27$ mA·cm^{-2}, FF ≈ 83%. and a maximum efficiency of ~7.65%, primarily limited by moderate absorption strength and defect-assisted recombination. Multilayer configurations indicate that geometric optimization can significantly enhance projected efficiency, approaching ~19–20% under idealized conditions. Although vacancy engineering extends visible-light absorption. the intrinsic indirect band-gap character constrains the ultimate photovoltaic performance of $ZnTiO_3$.

Keywords: $ZnTiO_3$ perovskite; oxygen vacancies; density functional theory (DFT); SCAPS-1D simulation; perovskite oxides; bandgap narrowing; photovoltaic performance

Table S1. Optimized fractional atomic coordinates of the cubic $ZnTiO_3$ supercell

Number	Atom	X-axis	Y-axis	Z-axis
1	Ti	0.1667	0.1667	0.1667
2	Ti	0.5000	0.1667	0.1667
3	Ti	0.8333	0.1667	0.1667
4	Ti	0.1667	0.5000	0.1667
5	Ti	0.5000	0.5000	0.1667
6	Ti	0.8333	0.5000	0.1667
7	Ti	0.1667	0.8333	0.1667
8	Ti	0.5000	0.8333	0.1667
9	Ti	0.8333	0.8333	0.1667
10	Ti	0.1667	0.1667	0.5000
11	Ti	0.5000	0.1667	0.5000
12	Ti	0.8333	0.1667	0.5000
13	Ti	0.1667	0.5000	0.5000
14	Ti	0.5000	0.5000	0.5000
15	Ti	0.8333	0.5000	0.5000
16	Ti	0.1667	0.8333	0.5000
17	Ti	0.5000	0.8333	0.5000
18	Ti	0.8333	0.8333	0.5000
19	Ti	0.1667	0.1667	0.8333
20	Ti	0.5000	0.1667	0.8333
21	Ti	0.8333	0.1667	0.8333
22	Ti	0.1667	0.5000	0.8333
23	Ti	0.5000	0.5000	0.8333
24	Ti	0.8333	0.5000	0.8333
25	Ti	0.1667	0.8333	0.8333
26	Ti	0.5000	0.8333	0.8333
27	Ti	0.8333	0.8333	0.8333
1	Zn	0.0000	0.0000	0.0000
2	Zn	0.3333	0.0000	0.0000
3	Zn	0.6667	0.0000	0.0000
4	Zn	0.0000	0.3333	0.0000
5	Zn	0.3333	0.3333	0.0000
6	Zn	0.6667	0.3333	0.0000
7	Zn	0.0000	0.6667	0.0000
8	Zn	0.3333	0.6667	0.0000
9	Zn	0.6667	0.6667	0.0000
10	Zn	0.0000	0.0000	0.3333
11	Zn	0.3333	0.0000	0.3333
12	Zn	0.6667	0.0000	0.3333
13	Zn	0.0000	0.3333	0.3333
14	Zn	0.3333	0.3333	0.3333
15	Zn	0.6667	0.3333	0.3333
16	Zn	0.0000	0.6667	0.3333
17	Zn	0.3333	0.6667	0.3333
18	Zn	0.6667	0.6667	0.3333
19	Zn	0.0000	0.0000	0.6667
20	Zn	0.3333	0.0000	0.6667
21	Zn	0.6667	0.0000	0.6667
22	Zn	0.0000	0.3333	0.6667
23	Zn	0.3333	0.3333	0.6667
24	Zn	0.6667	0.3333	0.6667

25	Zn	0.0000	0.6667	0.6667
26	Zn	0.3333	0.6667	0.6667
27	Zn	0.6667	0.6667	0.6667
1	O	0.0000	0.1667	0.1667
2	O	0.1667	0.0000	0.1667
3	O	0.1667	0.1667	0.0000
4	O	0.3333	0.1667	0.1667
5	O	0.5000	0.0000	0.1667
6	O	0.5000	0.1667	0.0000
7	O	0.6667	0.1667	0.1667
8	O	0.8333	0.0000	0.1667
9	O	0.8333	0.1667	0.0000
10	O	0.0000	0.5000	0.1667
11	O	0.1667	0.3333	0.1667
12	O	0.1667	0.5000	0.0000
13	O	0.3333	0.5000	0.1667
14	O	0.5000	0.3333	0.1667
15	O	0.5000	0.5000	0.0000
16	O	0.6667	0.5000	0.1667
17	O	0.8333	0.3333	0.1667
18	O	0.8333	0.5000	0.0000
19	O	0.0000	0.8333	0.1667
20	O	0.1667	0.6667	0.1667
21	O	0.1667	0.8333	0.0000
22	O	0.3333	0.8333	0.1667
23	O	0.5000	0.6667	0.1667
24	O	0.5000	0.8333	0.0000
25	O	0.6667	0.8333	0.1667
26	O	0.8333	0.6667	0.1667
27	O	0.8333	0.8333	0.0000

Table S2. Optimized fractional atomic coordinates of the hexagonal $ZnTiO_3$ supercell

Number	Atom	X-axis	Y-axis	Z-axis
1	O	0.8396	0.1791	0.2629
2	O	0.7928	0.0601	0.0380
3	O	0.9212	0.2131	0.6348
4	O	0.8957	0.0565	0.3997
5	O	0.3631	0.0747	0.3442
6	O	0.3223	0.2071	0.0652
7	O	0.4526	0.1365	0.6752
8	O	0.2956	0.0716	0.9407
9	O	0.3877	0.2479	0.6880
10	O	0.5063	0.0124	0.2628
11	O	0.4596	0.1432	0.0381
12	O	0.5878	0.0464	0.6349
13	O	0.5623	0.1397	0.3997
14	O	0.0299	0.1582	0.3441
15	O	0.9889	0.0406	0.0650
16	O	0.1193	0.2199	0.6751
17	O	0.9622	0.1551	0.9405
18	O	0.0543	0.0814	0.6880

19	O	0.1729	0.0957	0.2628
20	O	0.1262	0.2267	0.0378
21	O	0.2545	0.1296	0.6351
22	O	0.2290	0.2230	0.3997
23	O	0.6965	0.2415	0.3442
24	O	0.6555	0.1239	0.0650
25	O	0.7860	0.0533	0.6751
26	O	0.6289	0.2384	0.9405
27	O	0.7210	0.1647	0.6880
28	O	0.8396	0.4291	0.2629
29	O	0.7928	0.3101	0.0380
30	O	0.9212	0.4631	0.6348
31	O	0.8957	0.3065	0.3997
32	O	0.3631	0.3247	0.3442
33	O	0.3223	0.4571	0.0652
34	O	0.4526	0.3865	0.6752
35	O	0.2956	0.3216	0.9407
36	O	0.3877	0.4979	0.6880
37	O	0.5063	0.2624	0.2628
38	O	0.4596	0.3932	0.0381
39	O	0.5878	0.2964	0.6349
40	O	0.5623	0.3897	0.3997
41	O	0.0299	0.4082	0.3441
42	O	0.9889	0.2906	0.0650
43	O	0.1193	0.4699	0.6751
44	O	0.9622	0.4051	0.9405
45	O	0.0543	0.3314	0.6880
46	O	0.1729	0.3457	0.2628
47	O	0.1262	0.4767	0.0378
48	O	0.2545	0.3796	0.6351
49	O	0.2290	0.4730	0.3997
50	O	0.6965	0.4915	0.3442
51	O	0.6555	0.3739	0.0650
52	O	0.7860	0.3033	0.6751
53	O	0.6289	0.4884	0.9405
54	O	0.7210	0.4147	0.6880
55	O	0.8396	0.6791	0.2629
56	O	0.7928	0.5601	0.0380
57	O	0.9212	0.7131	0.6348
58	O	0.8957	0.5565	0.3997
59	O	0.3631	0.5747	0.3442
60	O	0.3223	0.7071	0.0652
61	O	0.4526	0.6365	0.6752
62	O	0.2956	0.5716	0.9407
63	O	0.3877	0.7479	0.6880
64	O	0.5063	0.5124	0.2628
65	O	0.4596	0.6432	0.0381
66	O	0.5878	0.5464	0.6349
67	O	0.5623	0.6397	0.3997
68	O	0.0299	0.6582	0.3441
69	O	0.9889	0.5406	0.0650
70	O	0.1193	0.7199	0.6751
71	O	0.9622	0.6551	0.9405

72	O	0.0543	0.5814	0.6880
73	O	0.1729	0.5957	0.2628
74	O	0.1262	0.7267	0.0378
75	O	0.2545	0.6296	0.6351
76	O	0.2290	0.7230	0.3997
77	O	0.6965	0.7415	0.3442
78	O	0.6555	0.6239	0.0650
79	O	0.7860	0.5533	0.6751
80	O	0.6289	0.7384	0.9405
81	O	0.7210	0.6647	0.6880
82	O	0.8396	0.9291	0.2629
83	O	0.7928	0.8101	0.0380
84	O	0.9212	0.9631	0.6348
85	O	0.8957	0.8065	0.3997
86	O	0.3631	0.8247	0.3442
87	O	0.3223	0.9571	0.0652
88	O	0.4526	0.8865	0.6752
89	O	0.2956	0.8216	0.9407
90	O	0.3877	0.9979	0.6880
91	O	0.5063	0.7624	0.2628
92	O	0.4596	0.8932	0.0381
93	O	0.5878	0.7964	0.6349
94	O	0.5623	0.8897	0.3997
95	O	0.0299	0.9082	0.3441
96	O	0.9889	0.7906	0.0650
97	O	0.1193	0.9699	0.6751
98	O	0.9622	0.9051	0.9405
99	O	0.0543	0.8314	0.6880
100	O	0.1729	0.8457	0.2628
101	O	0.1262	0.9767	0.0378
102	O	0.2545	0.8796	0.6351
103	O	0.2290	0.9730	0.3997
104	O	0.6965	0.9915	0.3442
105	O	0.6555	0.8739	0.0650
106	O	0.7860	0.8033	0.6751
107	O	0.6289	0.9884	0.9405
108	O	0.7210	0.9147	0.6880
1	Zn	0.6441	0.0725	0.3304
2	Zn	0.0839	0.1283	0.0658
3	Zn	0.2534	0.0290	0.6566
4	Zn	0.3108	0.1558	0.3307
5	Zn	0.7506	0.2116	0.0659
6	Zn	0.9200	0.1125	0.6566
7	Zn	0.9775	0.2393	0.3305
8	Zn	0.4173	0.0448	0.0659
9	Zn	0.5867	0.1958	0.6565
10	Zn	0.6441	0.3225	0.3304
11	Zn	0.0839	0.3783	0.0658
12	Zn	0.2534	0.2790	0.6566
13	Zn	0.3108	0.4058	0.3307
14	Zn	0.7506	0.4616	0.0659
15	Zn	0.9200	0.3625	0.6566
16	Zn	0.9775	0.4893	0.3305

17	Zn	0.4173	0.2948	0.0659
18	Zn	0.5867	0.4458	0.6565
19	Zn	0.6441	0.5725	0.3304
20	Zn	0.0839	0.6283	0.0658
21	Zn	0.2534	0.5290	0.6566
22	Zn	0.3108	0.6558	0.3307
23	Zn	0.7506	0.7116	0.0659
24	Zn	0.9200	0.6125	0.6566
25	Zn	0.9775	0.7393	0.3305
26	Zn	0.4173	0.5448	0.0659
27	Zn	0.5867	0.6958	0.6565
28	Zn	0.6441	0.8225	0.3304
29	Zn	0.0839	0.8783	0.0658
30	Zn	0.2534	0.7790	0.6566
31	Zn	0.3108	0.9058	0.3307
32	Zn	0.7506	0.9616	0.0659
33	Zn	0.9200	0.8625	0.6566
34	Zn	0.9775	0.9893	0.3305
35	Zn	0.4173	0.7948	0.0659
36	Zn	0.5867	0.9458	0.6565
1	Ti	0.8919	0.1068	0.1598
2	Ti	0.0202	0.9936	0.8292
3	Ti	0.4608	0.0621	0.5176
4	Ti	0.5586	0.1901	0.1597
5	Ti	0.6869	0.0769	0.8291
6	Ti	0.1275	0.1455	0.5176
7	Ti	0.2252	0.0234	0.1599
8	Ti	0.3535	0.1601	0.8293
9	Ti	0.7942	0.2288	0.5177
10	Ti	0.8919	0.3568	0.1598
11	Ti	0.0202	0.2436	0.8292
12	Ti	0.4608	0.3121	0.5176
13	Ti	0.5586	0.4401	0.1597
14	Ti	0.6869	0.3269	0.8291
15	Ti	0.1275	0.3955	0.5176
16	Ti	0.2252	0.2734	0.1599
17	Ti	0.3535	0.4101	0.8293
18	Ti	0.7942	0.4788	0.5177
19	Ti	0.8919	0.6068	0.1598
20	Ti	0.0202	0.4936	0.8292
21	Ti	0.4608	0.5621	0.5176
22	Ti	0.5586	0.6901	0.1597
23	Ti	0.6869	0.5769	0.8291
24	Ti	0.1275	0.6455	0.5176
25	Ti	0.2252	0.5234	0.1599
26	Ti	0.3535	0.6601	0.8293
27	Ti	0.7942	0.7288	0.5177
28	Ti	0.8919	0.8568	0.1598
29	Ti	0.0202	0.7436	0.8292
30	Ti	0.4608	0.8121	0.5176
31	Ti	0.5586	0.9401	0.1597
32	Ti	0.6869	0.8269	0.8291
33	Ti	0.1275	0.8955	0.5176

34	Ti	0.2252	0.7734	0.1599
35	Ti	0.3535	0.9101	0.8293
36	Ti	0.7942	0.9788	0.5177

Table S3. Total Free Energy of the studied $ZnTiO_3$ systems

System	Hexagonal Cell	Cubic cell
Supercell	-1243.929953	-883.9343251
Slab (101)	-1197.729802	-837.3808766
Slab with VO	-1193.166804	----
Slab with VO + U	-984.01638621	----
